# Improving the Expression of Recombinant Proteins in *E. coli* BL21 (DE3) under Acetate Stress: An Alkaline pH Shift Approach

**DOI:** 10.1371/journal.pone.0112777

**Published:** 2014-11-17

**Authors:** Hengwei Wang, Fengqing Wang, Wei Wang, Xueling Yao, Dongzhi Wei, Hairong Cheng, Zixin Deng

**Affiliations:** 1 Innovation & Application Institute (IAI), Zhejiang Ocean University, Zhoushan, China; 2 State Key Laboratory of Microbial Metabolism, School of Life Sciences and Biotechnology, Shanghai Jiao Tong University, Shanghai, China; 3 New World Institute of Biotechnology, State Key Laboratory of Bioreactor Engineering, East China University of Science and Technology, Shanghai, China; Scuola Internazionale Superiore di Studi Avanzati, Italy

## Abstract

Excess acetate has long been an issue for the production of recombinant proteins in *E. coli* cells. Recently, improvements in acetate tolerance have been achieved through the use of genetic strategies and medium supplementation with certain amino acids and pyrimidines. The aim of our study was to evaluate an alternative to improve the acetate tolerance of *E. coli* BL21 (DE3), a popular strain used to express recombinant proteins. In this work we reported the cultivation of BL21 (DE3) in complex media containing acetate at high concentrations. In the presence of 300 mM acetate, compared with pH 6.5, pH 7.5 improved cell growth by approximately 71%, reduced intracellular acetate by approximately 50%, and restored the expression of glutathione S-transferase (GST), green fluorescent protein (GFP) and cytochrome P450 monooxygenase (CYP). Further experiments showed that alkaline pHs up to 8.5 had little inhibition in the expression of GST, GFP and CYP. In addition, the detrimental effect of acetate on the reduction of 3-(4,5-dimethylthiazol-2-yl)-2,5-diphenyl tetrazolium bromide (MTT) by the cell membrane, an index of cellular metabolic capacity, was substantially alleviated by a shift to alkaline pH values of 7.5–8.0. Thus, we suggest an approach of cultivating *E. coli* BL21 (DE3) at pH 8.0±0.5 to minimize the effects caused by acetate stress. The proposed strategy of an alkaline pH shift is a simple approach to solving similar bioprocessing problems in the production of biofuels and biochemicals from sugars.

## Introduction

The use of glucose or glycerol as the carbon source in a complex medium may result in acetate accumulation at high levels, often up to 50–100 mM, particularly in high-density fermentations [1], [2]. In aqueous solution, the acetate anion (Ac^–^) exists in equilibrium with the undissociated acetic acid (HAc) and their relative quantities depend on pH. At 25°C the dissociation constant of acetic acid (pKa) is 4.75. Based on the Henderson-Hasselbalch equation, at pH 7.0 HAc is estimated to be 0.56 mM per 100 mM total acetate (Ac^–^ + HAc). As is well known, the HAc molecule is able to diffuse freely across the *E. coli* cell membrane and dissociates into Ac^–^ and H^+^ intracellularly [3]. Recently, Gimenez and coworkers found an acetate transporter protein in *E. coli*, ActP, with an affinity K_m_ for acetate of 5.4 µM, suggesting that it is an acetate scavenger that can enhance cellular survival under conditions of carbon source starvation [4]. However, when a complex medium is used to express recombinant proteins, carbon source starvation seldom occurs and acetate transport mainly depends on the free diffusion of HAc across the cell membrane [3]. In scale-up of bioprocesses from shake flasks to fermentors, acetate production tends to increase and is usually regarded as a cause of the decline in protein expression [2], [5], [6].

For decades many researchers have focused on strategies that reduce acetate accumulation. Briefly, the approaches developed involve process optimization and host organism modification [5], [6]. Eiteman and Altman suggested that *E. coli* cells produce acetate when they have surpassed a threshold value of the specific rate of glucose consumption and that only under glucose limitation is the specific growth rate directly related to acetate production [5]. Limiting the glucose concentration of the medium is regarded as a valid strategy for reducing acetate accumulation. Akesson and coworkers developed an automated glucose feeding strategy by controlling dissolved oxygen (DO) through manipulating the stirrer speed and successfully reduced acetate accumulation to less than 60 mg/L (1.0 mM) [7]. An alternative is to reduce glucose uptake of the host cells by genetic modification. Wong and coworkers disabled the phosphoenolpyruvate: sugar phosphotransferase system (PEP-PTS) by deleting the *pts*HI operon in wild-type *E. coli* strain GJT001 and found that in 2×LB broth with 2% glucose the mutant TC110 was able to grow quickly and produced much less acetate (9.1±6.6 mM in TC110 *vs.* 90.4±1.6 mM in GJT001) [8]. Lara and coworkers constructed an *E. coli* strain VH32 lacking PTS with a modified glucose transport system and found that strain VH32 cultured at glucose concentrations of up to 100 g/L produced a maximum concentration of only 2 g/L (33 mM) acetate, while its parental strain W3110 accumulated a maximum of 13.6 g/L (227 mM) acetate [9]. They thus demonstrated the possibility of a return to simple batch cultivations using the PTS-deficient strain instead of the traditional fed-batch cultivations that aim to avoid high glucose concentrations. Valgepea and coworkers studied changes in cultivations of *E. coli* (accelerostat cultivation with continuous change of specific growth rate and dilution-rate-stat cultivation with a smoothly changing environmental parameter) [10]. They found that the acetate-consuming capability of *E. coli* was dependent on the specific growth rate and decreased by 12-fold around the overflow switch growth rate of 0.27±0.02 h^−1^ [10]. Thus, they suggested a correlation between glucose-mediated cyclic-AMP receptor protein (cAMP-CRP) (that regulates the expression of acetyl-CoA synthetase transcribing the *acs*-*yjc*H-*act*P operon) repression and acetyl-CoA synthetase (Acs) down-regulation, which results in decreased assimilation of acetate produced by phosphotransacetylase (Pta), and disruption of the Pta-Acs node. The above studies shed light on a future when we can use a PTS-deficient and Acs-overexpressing *E. coli* strain in a simple batch cultivation with no need to restrict the glucose concentration to avoid acetate accumulation.

On the other hand, a few studies have turned to improving *E. coli* tolerance towards acetate because of its promising applications in biofuel and chemical production from lignocellulose feedstocks [11], [12]. Earlier, Han and coworkers found that addition of some amino acids at 0.5 g/L, in particular methionine, alleviated the inhibition of the specific growth rate of *E. coli* K-12 caused by acetate at up to 8 g/L [13]. Roe and coworkers studied the growth inhibition of *E. coli* K-12 caused by acetate in a defined medium based upon citrate-phosphate buffer at pH 6.0 and concluded that acetate caused the depletion of the intracellular methionine pool and the concomitant accumulation of the intermediate homocysteine, which was found to inhibit the growth of *E. coli* [14], [15]. In the methionine biosynthesis pathway, MetA (EC2.3.1.46, homocysteine O-succinyltransferase) is the first enzyme that catalyzes the transfer of succinate from succinyl-coenzyme A to L-homoserine [16], [17] and MetE (EC 2.1.1.14, cobalamin-independent homocysteine transmethylase) catalyzes the final step to form methionine from L-homocysteine [18], [19]. Mordukhova and coworkers mutated the MetA and MetE enzymes and found that the introduction of thermostable mutants of MetA and/or MetE to *E. coli* strains improved the cell growth in M9 glucose medium (pH 6.0) supplemented with 20 mM sodium acetate [20], [21]. Sandoval et al. tested the alleviating effect of specific amino acids and/or pyrimidine bases on the acetate toxicity and found that supplementation of four amino acids (arginine, methionine, threonine and lysine) and two pyrimidines (cytosine and uracil) to the minimal growth medium led to restoration of the specific growth rate at different levels [12]. Their results suggest that acetate inhibition might be meditated by a broad range of genes and pathways [12]. Chong and coworkers introduced random mutations to CRP in a host strain *E. coli* DH5α, which was constructed by knocking out its original *crp* gene, and isolated a mutant A2 with the greatest tolerance to acetate [22]. Further results showed that its specific growth rate was much higher than that of the control (0.083 *vs.* 0.016 h^−1^) when exposed to the stress of 15 g/L (183 mM) sodium acetate (NaAc) in M9 minimal medium [22].

*E. coli* BL21 (DE3) is a popular strain used for recombinant protein expression and its growth in complex media containing glucose or glycerol tends to result in acetate accumulation at different levels, depending on the specific strategy for process control. It is well documented that recombinant protein expression in *E. coli* cells is decreased by acetate accumulation [5], [6]. However, our previous study showed that acetate concentrations up to 200 mM had little effect on the protein expression of recombinant tissue plasminogen activator (rtPA) in this strain [2].

In this study, the acetate tolerance of *E. coli* BL21 (DE3) was further confirmed, and specific conditions for the cultivation of and protein expression in this strain were investigated in complex media at high acetate concentrations (up to 300 mM). A comparison of cell growth at pH 6.5 and pH 7.5 and intracellular acetate accumulation and recombinant protein expression of GST, CYP and GFP under acetate stress suggested that an alkaline pH shift would be an alternate approach for improving the tolerance to acetate. Further experiments showed that alkaline pH up to 8.5 supported bacterial growth well and inhibited the expressions of GST, CYP and GFP very little. In addition, we also studied the effects of up to 300 mM NaCl and NaAc on the MTT-reducing activity of the *E. coli* cell membrane to show the differences in inhibition of cell growth and protein production caused by salt and acetate stress. The alleviation of the detrimental effects of acetate on the performance of this strain by a shift to alkaline pH (7.5–8.5) suggests a simple approach to solving such bioprocessing problems.

## Materials and Methods

### Bacterial strains and plasmids

*E. coli* BL21 (DE3) (F^–^ *dcm ompT hsdS_B_* (r_B_^–^ m_B_^–^) *gal*) was used in all experiments. The strains *E. coli* BL21 (DE3), Origami (DE3) and DH5α and the plasmids pET-28a (+), pET-32a (+) and pRSET-B were purchased from Novagen, USA. Gene *gst* was from pET-4T-1 (Amersham, USA) and expressed glutathione S-transferase (GST, 26 kDa) using pET-32a(+). An enhanced green fluorescent protein (GFP, 28 kDa) and a cytochrome P450 monooxygenase (CYP, 44 kDa) were expressed using pET-28a(+). TAT-survivin (T34A) (12 kDa), a mutant of tumor suppressor protein, was expressed using pRSET-B [23]. The expressed GST was a soluble protein, the previously reported protein rtPA was in the form of inclusion bodies [2], and the expressed GFP, CYP and TAT-survivin (T34A) were in both forms.

### Media and conditions

Buffered YT medium (BYT) contained 5 g/L yeast extract (Oxoid, UK), 10 g/L tryptone (Oxoid, UK), 9 g/L Na_2_HPO_4_•12H_2_O and 1 g/L KH_2_PO_4_, and its pH was set at 7.0±0.2 before sterilization. Media 2×BYT and 4×BYT contained two- and four-fold BYT ingredients. Other stock solutions, including sterile water, 250 g/L NaOH, 25% phosphoric acid (25 mL of 85% phosphoric acid/100 mL, v/v), 600 mM NaAc, 600 mM NaCl, 500 g/L glycerol and 500 g/L glucose, were autoclaved seperately. BYT-glycerol medium contained 10 g/L glycerol, and BYT-glucose medium contained 10 g/L glucose. YTA medium contained 10 g/L yeast extract (Oxoid, UK), 10 g/L tryptone (Oxoid, UK), 33 g/L (NH4)_2_SO_4_, 4.5 g/L Na_2_HPO_4_•12H_2_O and 0.5 g/L KH_2_PO_4_. All media and stock solutions were autoclaved at 121°C for 15 min.

Other media in our study were prepared using the above stock solutions and incubated at 37°C for at least 1 h before inoculation. BYT-glycerol medium (pH 7.0) was used for preculture preparation, unless otherwise specified. All precultures of *E. coli* cells harboring various plasmids were grown for 10 h before inoculation. To grow *E. coli* strains harboring plasmids, 100 mg/L ampicillin was used in the case of pET-32a(+) and pRSET-B, and 50 mg/L kanamycin in the case of pET-28a(+). To express recombinant proteins, 1 mM isopropyl β-D-1-thiogalactopyranoside (IPTG) was used. Solutions of 250 g/L NaOH and 25% (v/v) phosphoric acid were used to adjust pH in situ during cultivation. To reduce the effect of sampling on cell growth, all shake flask experiments were conducted on a 30 mm orbital shaker at 200 rpm (Hualida, China) in an incubation room at 37°C.

### TAT-survivin (T34A) expression under acetate stress in shake flasks and bioreactors

In the case of shake flask cultivation, TAT-survivin (T34A) expression was carried out in YTA-glucose medium containing 0, 50, 100, 200 and 300 mM NaAc at pH 7.5. Glucose was used to lower auto-induction [24]. At inoculation, 1 mL of precultured *E. coli* BL21 (DE3) harboring pRSET-B-TAT-survivin (T34A) was inoculated into 50 mL medium in a 250-mL Erlenmeyer flask. Baterial cells were cultivated for 4 h and induced by 1 mM IPTG for 4 h. Sodium dodecyl sulfate polyacrylamide gel electrophoresis (SDS-PAGE) was performed as described by Laemmli using a separating gel of 12.5% acrylamide and approximately 15 µg of cellular proteins per lane [25].

In the case of fermentor cultivation, the inoculum volume was 20 mL of preculture per liter of medium. The fed-batch cultivations were carried out in a 4-L BIOSTAT B fermentor (B. Braun Biotech, Germany) and a 30-L fermentor (Guoqiang Bioengineering Equipment, Shanghai, China) with initial volumes of 3.7 L and 25 L, respectively. Medium glucose was monitored offline using a GOD-POD kit (Kexin Biotech, Shanghai, China) according to the manufacturer's instructions and was maintained at 10±5 g/L by using a glucose stock solution of 500 g/L. The temperature was maintained at 37°C, and the pH was controlled at 7.5±0.2 using 25% NaOH solution. The dissolved oxygen (DO) was monitored using a polarographic oxygen electrode and the airflow rate was kept at 2.0 L/min per liter broth.

### Cell growth of *E. coli* BL21 (DE3) under acetate stress

The experiments were conducted in the BYT-glycerol medium containing 0, 50, 100, 200 and 300 mM NaAc or NaCl at pH 6.5 and pH 7.5 To start the cultivation, *E. coli* BL21 (DE3) preculture grown for 10 h was inoculated into 20 mL of BYT-glycerol medium in a 250-mL Erlenmeyer flask at 37°C. Samples were taken in situ every 30 min to monitor cell growth by measurement of the optical density (OD) at 600 nm (OD_600_). To avoid oxygen limitation on cell growth, the initial OD_600_ was set at 0.15∼0.2. The specific growth rate (h^−1^) was calculated according to the formula X_2_ = X_1_·e^μΔt^ using OD_600_ values from the first 3 h of cultivation. Experiments were conducted in duplicate for each NaAc and NaCl gradient at each pH.

To determine the dry cell weight (DCW), 10 mL of culture were collected by centrifugation at 4000 *g* for 5 min, washed with deionized water and dried at 115°C for about 24 h to constant weight. OD_600_ of 1.0 corresponded to approximately 0.385 g of DCW per liter culture of *E. coli*.

### Accumulation of intracellular acetate in *E. coli* cells under acetate stress

The experiments were performed in the BYT- glycerol medium containing 50, 100, 200 and 300 mM NaAc at pH 6.5 and pH 7.5. *E. coli* BL21 (DE3) cells were first grown for 10 h, inoculated into 100 mL of BYT-glycerol medium (pH 7.0) in a 500-mL Erlenmeyer flask, cultivated to an OD_600_ of approximately 2.5 and collected by centrifugation at 8000 *g* for 5 min at room temperature. The cells were then resuspended thoroughly in 50-mL centrifuge tubes by vigorous vortexing in the BYT-glycerol medium (pH 6.5 and 7.5). To prepare the acetate-stressed medium, 4×BYT solution was adjusted to pH 6.5 and pH 7.5 and mixed with 600 mM NaAc solution of the same pH, 500 g/L glycerol solution, and the resuspended cells in a 250-mL Erlenmeyer flask. Sterile water was then added to adjust the final volume to 20 mL. All stock solutions and flasks were incubated at 37°C for at least 1 h in advance. In order to obtain a sufficient amount of cells for the determination of intracellular acetate concentration, the *E. coli* cells were adjusted to an OD_600_ of 5.0 and 1.0-mL samples were taken at 2, 10 and 40 min.

To collect *E. coli* cells, 1.0 mL of sample was centrifuged in a 1.5-mL tube at 10,000 *g* for 25 sec, the supernatant medium was pipetted away, the cell pellet together with the tube bottom was separated by cutting the tube, and the residual extracellular liquid medium in the pellet was then removed by absorbance using tissue paper. Using the above sampling protocol, the extracellular medium remaining was estimated to be 2.5±0.5 µL determined by trypan blue dye exclusion [26]. The intracellular acetate was extracted according to the method of Taymaz-Nikerel et al. with some modification [27]. In brief, the above cell pellets together with the tube bottom were quickly transferred into 1.0 mL of 80% hot ethanol (90°C) in a 5-mL centrifuge tube on a water bath and incubated for 5 min. After cooling on ice for 5 min, the extracts obtained were evaporated to dryness at 45°C for 60 min in a vacuum evaporation system RapidVap (Labconco, USA) and stored at −80°C for further analysis. It shoud be noted that, to reduce the sampling time, the orbital shaker, the centrifuge and the water bath were all put together in the same incubation room set at 37°C; and to avoid leakage of intracellular acetate, samples were subjected to direct extraction of acetate without any cell washing or quenching steps. The intracellular acetate concentrations were equal to the concentrations of total acetate minus those of the extracellular acetate contained in the residual medium.

Acetate determination was performed on an ion chromatography system (Dionex ICS 1500) equipped with a guard column IonPac AG11-HC (4×50 mm) and an analytical column IonPac AS11-HC (4×250 mm). To dissolve the above cell extracts, 1000 µL of ultrapure water (18.2 MΩ•cm at 25°C) were added to the 5-mL centrifuge tubes, mixed by vortexing and allowed to stand for 10 min. Then, the samples were centrifuged at 5000 *g* for 10 min and the supernatants were further removed by 0.22-µm filtration. Potassium hydroxide (KOH) was used as the eluent (5 mM from 0 to 8 min, 30 mM from 8 to 16 min and 5 mM end program) at a flowrate of 1.0 mL/min and the column temperature was set at 30°C.

### pH dependence of bacterial growth and recombinant protein expression

For bacterial growth, *E. coli* BL21 (DE3) cells were grown in 20 mL of BYT-glycerol medium at pH 6.5, 7.0, 7.5, 8.0 and 8.5 in 250-mL Erlenmeyer flasks at 37°C. Experiments were performed in duplicate. To avoid side-effects of sterilization at alkaline pH, BYT medium was autoclaved at pH 6.5 and then adjusted to set-up pH values. For recombinant protein expression, GST, CYP and GFP were expressed by *E. coli* BL21 (DE3) in the BYT-glycerol medium at pH 6.5, 7.5 and 8.5 under the same conditions. At inoculation, 100 mg/L of ampicillin was used in the case of pET-32a(+)-GST and 50 mg/L kanamycin was used in the case of pET-28a(+)-GFP and pET-28a(+)-CYP. Baterial cells were cultivated for 2 h and induced by 1 mM IPTG for 4 h. The medium pH decreased during the 6-h cultivation and was adjusted in situ using 20–100 µL of 25% NaOH solution.

### Recombinant protein expression under acetate stress

Recombinant proteins GST, CYP and GFP were expressed by *E. coli* BL21 (DE3) in BYT-glycerol medium containing 300 mM NaAc at pH 6.5 and 7.5; 300 mM NaCl was used as a control. Other conditions were the same as in the experiments of recombinant protein expression at different pHs.

### Effect of alkaline pH shift on *E. coli* cell growth under acetate stress at various temperatures

Three *E. coli* strains, BL21 (DE3), Origami (DE3) and DH5α were used in the experiment. BYT-glycerol medium (pH 7.0) was used for the preculture preparation of *E. coli* BL21 (DE3) and DH5α, and BYT-glycerol medium (pH 7.0) supplemented with 50 mg/L kanamycin and 50 mg/L tetracycline was used for the preculture preparation of *E. coli* Origami (DE3). Specific growth rates were determined at 20, 25, 30, 35 and 40°C in the BYT-glycerol medium containing 300 mM NaAc at pH 6.5 and pH 8.0, using 300 mM NaCl as a control. To start the cultivation, 500 µL of *E. coli* preculture grown for 10 h was inoculated into 20 mL of BYT-glycerol medium containing NaAc or NaCl in a 250-mL Erlenmeyer flask and pre-incubated for 30 min to allow temperature recovery. Samples were then taken in situ every 1 h to monitor cell growth. To avoid oxygen limitation on cell growth, OD_600_ values from the first 3 h of cultivation were used. Experiments were performed in duplicate for each temperature.

### pH dependence of *E. coli* cellular viability under acetate stress

*E. coli* cellular viability was determined according to the MTT method as previously described [28]. The experiments were conducted in BYT- glycerol medium containing 0, 50, 100 and 300 mM NaAc or NaCl at pH 6.5, 7.0, 7.5 and 8.0. *E. coli* BL21 (DE3) cells were first grown for 10 h, then inoculated into 20 mL of BYT-glycerol medium (pH 7.0) in a 250-mL Erlenmeyer flask and cultivated for 4–5 h to the mid-exponential growth phase (to an OD_600_ of approximately 2.0). The cells were diluted to an OD_600_ of 0.100 using 2×BYT-glycerol solution (pH 6.5, 7.0, 7.5 and 8.0) before MTT assay. Then, 100 µL of cell dilution were mixed with 100 µL of NaAc or NaCl solution of the same pH in 1.5-mL centrifuge tubes. To start MTT reduction, 20 µL of 5 g/L MTT solution were added to the above mixtures and the tubes were incubated at 37°C for 20 min. The complex of formazan crystals and cells was collected by centrifugation and dissolved in alkaline dimethyl sulfoxide (DMSO) [29]. One MTT reduction unit (MRU) was defined as an absorbance of 1.0 at 540 nm in 20 min and the MTT reduction activity was expressed as MRU per OD_600_ per mL culture.

## Results

### Expression of recombinant protein TAT-survivin (T34A) by *E. coli* BL21 (DE3) at high concentrations of acetate

**Figure 1:**
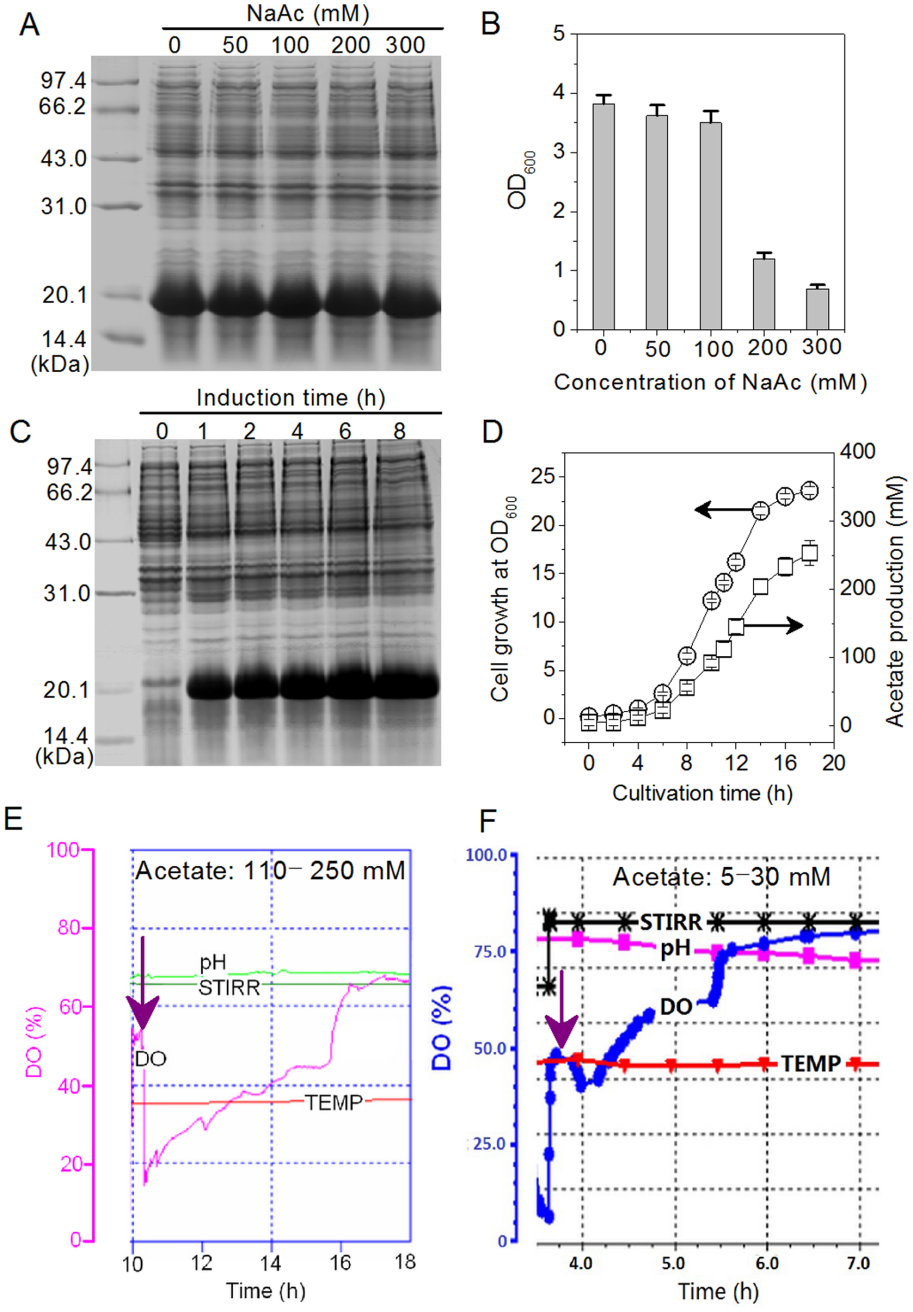
TAT-survivin (T34A) expression in *E. coli* BL21 (DE3) under acetate stress in shake flasks and bioreactors. A) and B), TAT-survivin (T34A) expression and cell growth of *E. coli* BL21 (DE3) under different conditions of acetate stress in shake flasks. The experiments were conducted in 50 mL of YTA-glucose medium (pH 7.5). C) and D), The profiles of TAT-survivin (T34A) expression, cell growth and acetate production in a 30-L bioreactor. The strategy of maintaining glucose at 10±5 g/L led to a constant increase of medium acetate from 10 h. E) The DO profile of the 30-L bioreactor cultivation from 10 h when IPTG was added (acetate: 110–250 mM). F) The DO profile of the 4-L bioreactor cultivation from 4 h when IPTG was added (acetate: 5–30 mM). In both bioreactors *E. coli* BL21 (DE3) cells were cultivated in YTA medium, but induced at different levels of acetate.

Our previous study demonstrated that *E. coli* BL21 (DE3) cells could produce acetate of up to 250 mM even on a medium containing glycerol as the sole carbon source and that the accumulation of medium acetate was not responsible for poor rtPA expression in the scale-up from shake flasks to fermentors [2]. This discovery seemed inconsistent with previous reports on the inhibition by acetate of recombinant protein expression [5], [6], [30]. In this experiment, TAT-survivin (T34A) expression by *E. coli* BL21 (DE3) was further examined on acetate of up to 300 mM, a concentration not reported in previous studies. Figure 1A shows that in shake flasks, TAT-survivin (T34A) expression stayed at the same level of approximately 28% by SDS-PAGE in the YTA-glucose medium containing up to 300 mM NaAc. But NaAc at 200 and 300 mM greatly inhibited bacterial growth as indicated by a 70∼80% drop in OD_600_ during cultivation, compared with the control without exogenous NaAc (Figure 1B). In the case of the 30-L fermentor, TAT-survivin (T34A) expression at 4–8 h of induction was at the same level as in shake flasks (Figure 1C), although medium acetate increased from 110 mM to 250 mM over the induction period from 10 h to 18 h (Figure 1D). Bacterial growth slowed down greatly from 0.31∼0.48 h^−1^ at 6–10 h to 0.01∼0.03 h^−1^ at 16–18 h (Figure 1D). These results demonstrated that acetate at up to 300 mM did not inhibit TAT-survivin (T34A) expression but did inhibit cell growth of *E. coli* BL21 (DE3).

We also observed that the cellular respiration activity declined during the induction (Figure 1E). From 10 h into induction, the DO in the 30-L fermentor continued to increase from 18% to 65% under constant oxygen supply conditions when the stirring speed and the airflow rate remained unchanged. In contrast, the DO in a 4-L fermentor increased from 4 h into induction at 5 mM acetate, suggesting that it was the protein expression that caused the DO to rise during induction, similar to the case of rtPA expression [2].

### *E. coli* cell growth on acetate at pH 6.5 and 7.5

**Figure 2:**
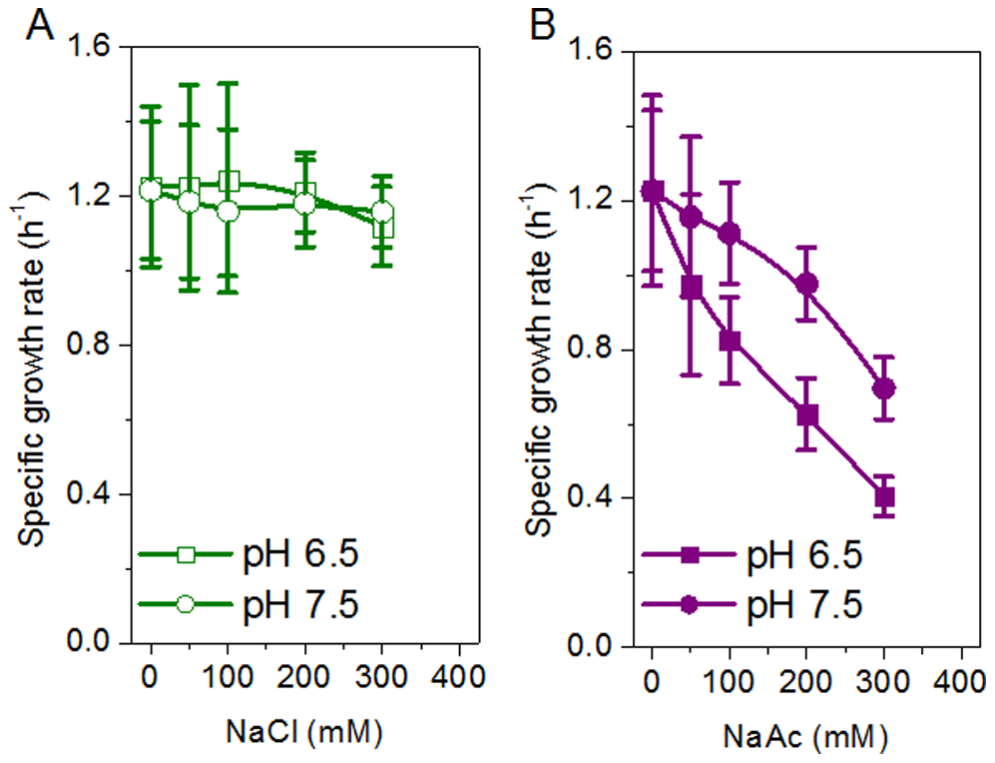
Effect of pH on cell growth of *E. coli* BL21 (DE3) in BYT-glycerol medium containing NaCl (A) and acetate (B).

Bacterial growth of *E. coli* cells on NaAc was determined at pH 6.5 and 7.5 (Figure 2). The results show that neither pH had an effect on cell growth for 0–300 mM NaCl in controlled cultivation and the specific growth rate remained constant at 1.20±0.22 h^−1^ (Figure 2A). But there was a significant improvement in cell growth rate at pH 7.5 on the NaAc-containing medium (Figure 2B). For example, at 300 mM NaAc, the specific cell growth rate increased by approximately 71% from 0.41±0.05 h^−1^ at pH 6.5 to 0.70±0.10 h^−1^ at pH 7.5.

### Intracellular acetate accumulation in *E. coli* cells at pH 6.5 and pH 7.5

**Figure 3:**
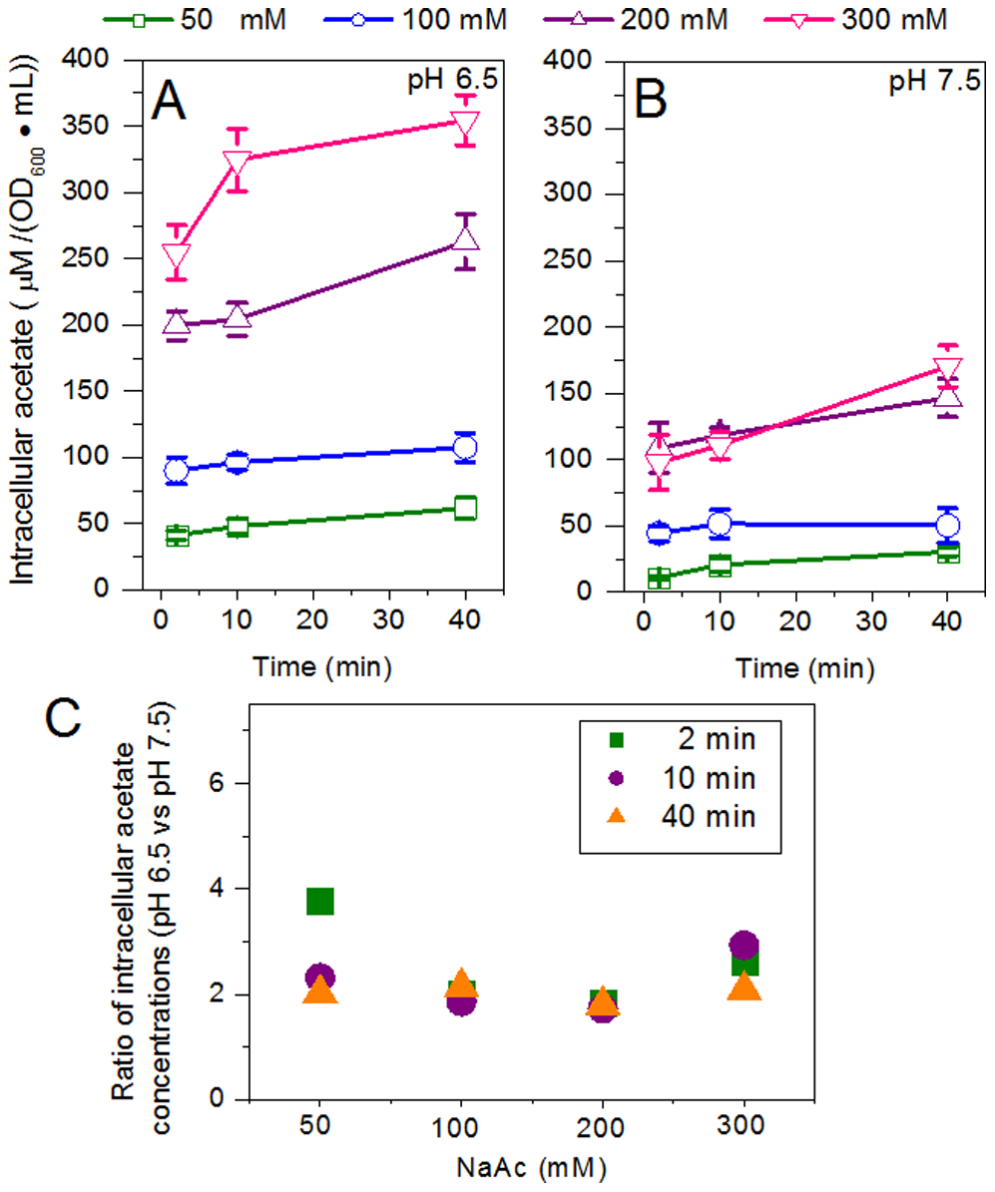
Intracellular acetate accumulation in *E. coli* BL21 (DE3) cells under acetate stress at 50, 100, 200 and 300 mM in BYT-glycerol medium at pH 6.5 (A) and 7.5 (B). C) Ratio of intracellular acetate concentration at pH 6.5 *vs.* pH 7.5.

Figure 3 shows the intracellular acetate accumulation at pH 6.5 and pH 7.5 in the BYT-glycerol medium containing 50–300 mM NaAc. The intracellular acetate concentration increased over the 40-min incubation in both cases, but was higher for each of the NaAc concentrations at pH 6.5 than that at pH 7.5 (Figure 3A and 3B). At pH 6.5, at the end of incubation, the intracellular acetate concentration reached approximately 62, 108, 263 and 355 µM/(OD_600_ • mL) on 50, 100, 200 and 300 mM NaAc, respectively. In the case of pH 7.5, it was 30, 50, 147 and 171 µM/(OD_600_ • mL). The intracellular acetate concentrations at pH 6.5 were approximately 2∼3-fold those at pH 7.5 over the 40-min incubation (Figure 3C).

If 1.6 µL/mg DCW is taken as the intracellular volume of *E. coli* cells as determined by Roe et al. [14], at the end of incubation on 50, 100, 200 and 300 mM NaAc, the absolute intracellular acetate concentrations were approximately 49, 81, 239 and 278 mM at pH 7.5 and 101, 175, 427 and 576 mM at pH 6.5. The data show that the intracellular acetate concentrations were approximately the same as those of extracellular NaAc at pH 7.5 and they were twice those at pH 6.5. The results from Figure 2 and 3 suggest the possibility of increasing acetate tolerance by a shift to alkaline pH to improve cell growth and reduce intracellular acetate accumulation.

### Effect of alkaline pH on cell growth and recombinant protein expression

**Figure 4:**
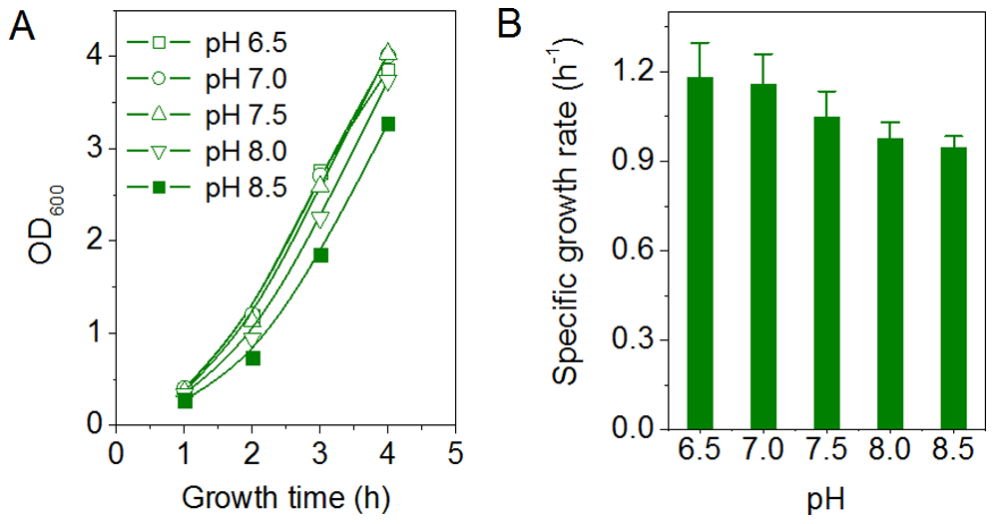
Effect of alkaline pHs on the cell growth of *E. coli* BL21 (DE3). A) Curves of cell growth over the 4-h cultivation at pH 6.5, 7.0, 7.5, 8.0 and 8.5. B) The specific cell growth rates at various pHs. The specific cell growth rates were determined from the cell growth curves over the first 3 h of cultivation in 20 mL of BYT-glycerol medium in 250-mL shake flasks.

**Figure 5:**
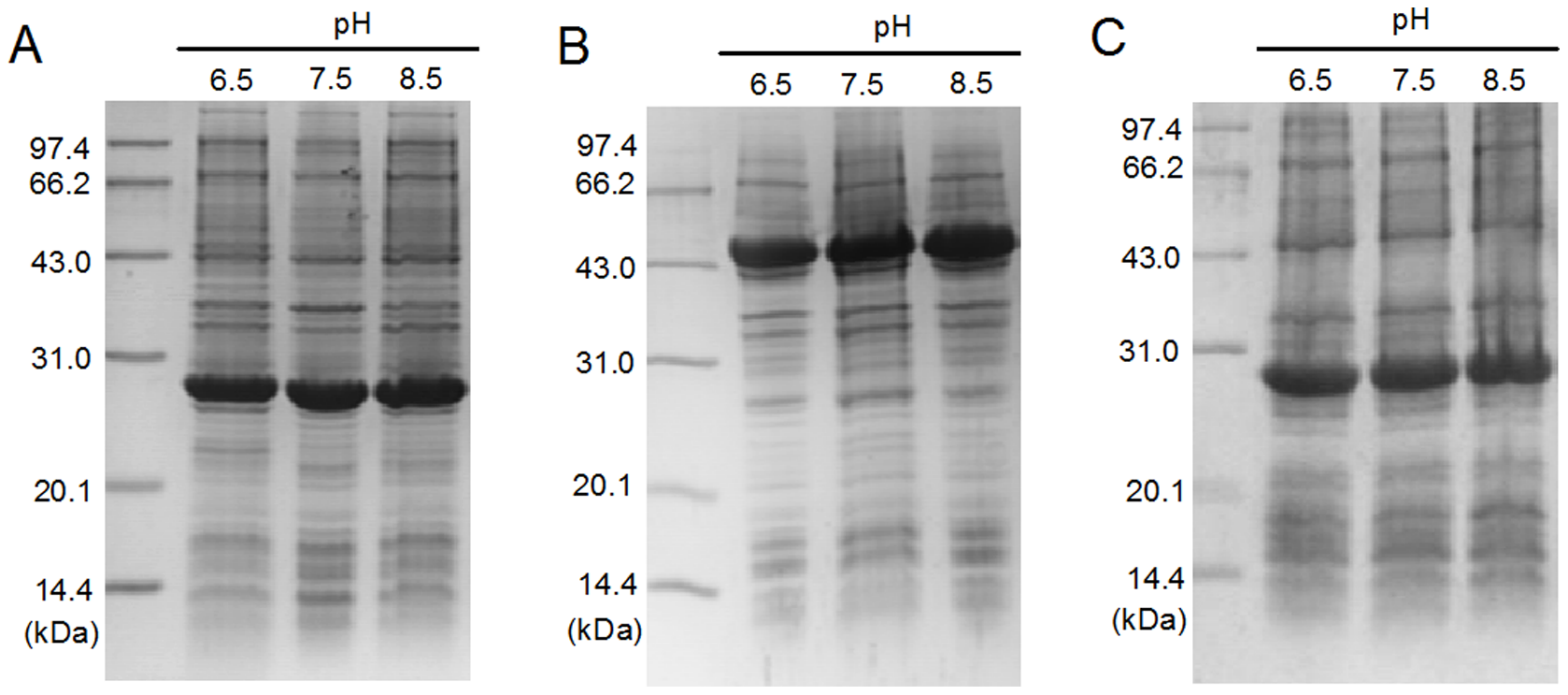
Effect of alkaline pHs on recombinant protein expression by *E. coli* BL21 (DE3). A) GST, B) CYP and C) GFP. The protein expression was conducted at pH 6.5, 7.5 and 8.5 in 20 mL of BYT-glycerol medium in 250-mL shake flasks as described in Methods and Materials.

To investigate cell growth at alkaline pH, *E. coli* BL21 (DE3) cells were grown in BYT-glycerol medium at various pH values from 6.5 to 8.5 (Figure 4). The results showed that all pH values were suitable for cell growth (Figure 4A), but at pH 7.5–8.5 the cell growth rate was lower compared with pH 6.5–7.0 and, in particular, an obvious delay in cell growth by approximately 21% was observed at pH 8.5 (Figure 4A and B). The expressions of recombinant proteins, GST, CYP and GFP, were also investigated at pH 6.5, 7.5 and 8.5. The results showed that alkaline pH (7.5 and 8.5) did not inhibit protein expression, as judged by SDS-PAGE scanning (Figure 5).

**Figure 6:**
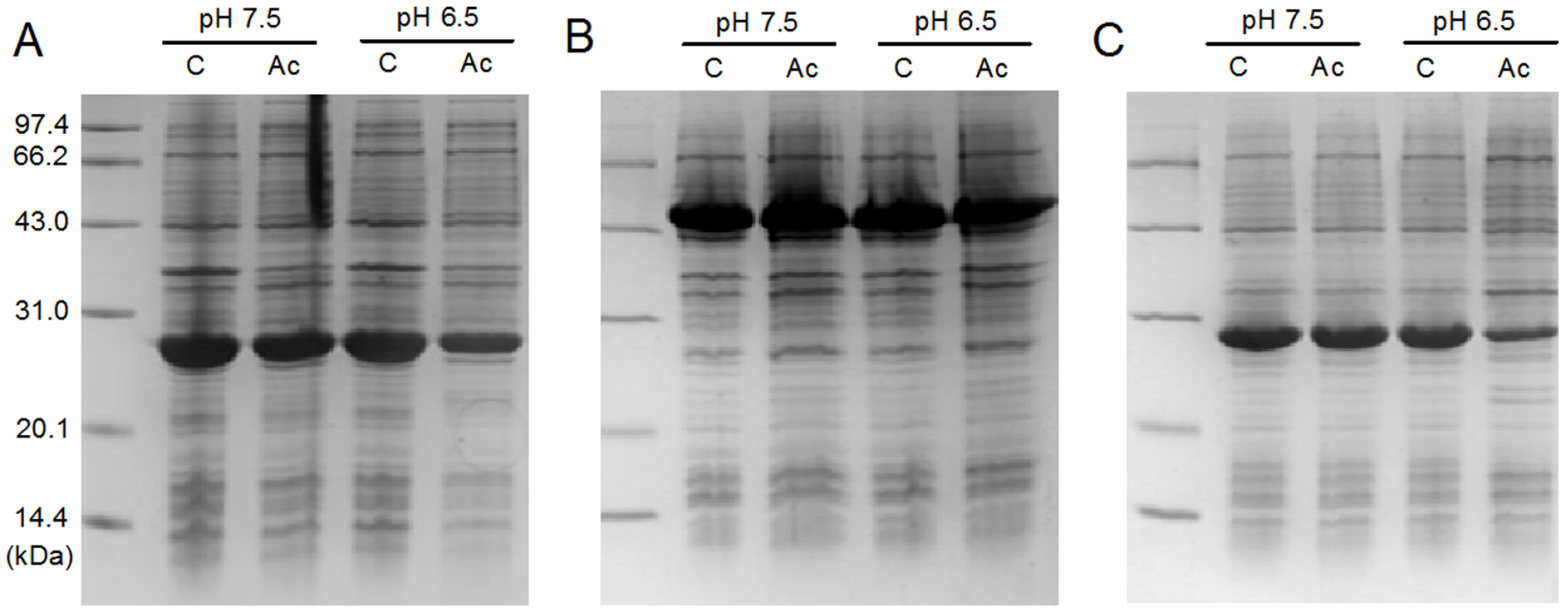
Comparison of the pH effect on recombinant protein expression by *E. coli* BL21 (DE3) grown on 300 mM NaAc. A) GST, B) CYP and C) GFP. The protein expression was conducted at pH 6.5 and 7.5 in 20 mL of BYT-glycerol medium in 250-mL shake flasks. C, BYT-glycerol medium containing 300 mM NaCl as control; Ac, BYT-glycerol medium containing 300 mM NaAc.

The use of alkaline pH in *E. coli* cultivation would be more valuable if it could alleviate the negative effect of acetate stress on the expression of recombinant proteins. We investigated the effect of pH 7.5 on the expression of GST, CYP and GFP by *E. coli* BL21 (DE3) grown on 300 mM NaAc in BYT-glycerol medium (Figure 6). At pH 6.5, the addition of 300 mM NaAc significantly decreased the expression levels of GST and GFP, by more than 60% compared with the control medium containing 300 mM NaCl (Figure 6A and 6C). At pH 7.5, 300 mM NaAc did not inhibit the expression of GST and GFP (Figure 6A and C). However, the drop in CYP expression on 300 mM NaAc at pH 6.5 was estimated at only 15% (Figure 6B).

**Figure 7:**
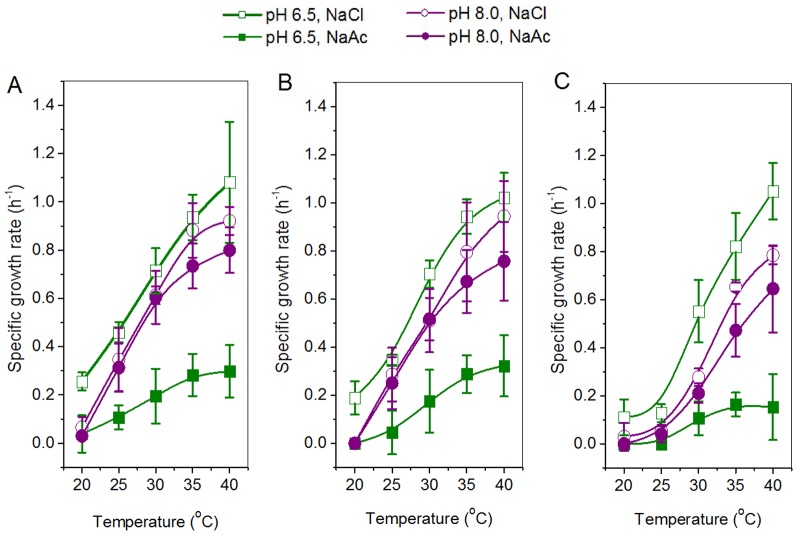
Alleviation of acetate-caused cell growth inhibition by alkaline pH shift from pH 6.5 to pH 8.0 at various temperatures. *E. coli* strains A) BL21 (DE3), B) Origami (DE3) and C) DH5α were tested. Specific growth rates were determined in 20 mL of BYT-glycerol medium containing 300 mM NaCl or NaAc in 250-mL shake flasks.

Alleviation of the acetate-caused cell growth inhibition by alkaline pH shift was also observed in the case of *E. coli* Origami (DE3) and DH5α. Under the stress of 300 mM NaAc, the specific growth rates of the three strains tested at pH 8.0 were remarkably higher than those at pH 6.5, especially at 30–40°C (Figure 7A, 7B and 7C). For example, the specific growth rate of *E. coli* Origami (DE3) at 30°C was approximately 0.52 h^−1^ at pH 8.0 but 0.17 h^−1^ at pH 6.5 (Figure 7B). Besides, an increase in cultivation temperature from 20 to 40°C greatly improved the cellular growth rates of the three strains under the stress of 300 mM NaCl and NaAc (Figure 7A, 7B and 7C). But *E. coli* DH5α was more sensitive to low temperatures below 30°C and its specific growth rates at pH 8.0 under the stress of 300 mM NaAc were close to 0 at 25°C, while those of *E. coli* BL21 (DE3) and Origami (DE3) were estimated at 0.25∼0.31 h^−1^ (Figure 7A and 7B vs. 7C). *E. coli* strains BL21 (DE3) and Origami (DE3) showed quite similar growth profiles under the conditions in our experiments (Figure 7A and 7B). However, it does not mean that, addition of a plasmid or gene fragment into *E. coli* cells will not affect its acetate resistance. The study by Mordukhova and Pan showed that introduction of the plasmids associated with methionine metabolism increased the resistance of *E. coli* host cells to acetate [21].

### Effect of alkaline pH on the restoration of cellular viability under acetate stress

MTT, as a soluble substrate, is reduced by electrons produced via respiration in the bacterial membrane into insoluble purple crystals of formazan. As the reaction proceeds, formazan crystals are gradually deposited on the surface of the bacterial cells, and form crystal-cell complexes [28], [29]. The crystal-cell complexes formed were collected, dissolved in alkaline DMSO and measured using a colorimetric method [28], [31]. MTT reduction activity was thus used as an index to determine the extent of electron production by the *E. coli* cell membrane [28], [31].

**Figure 8:**
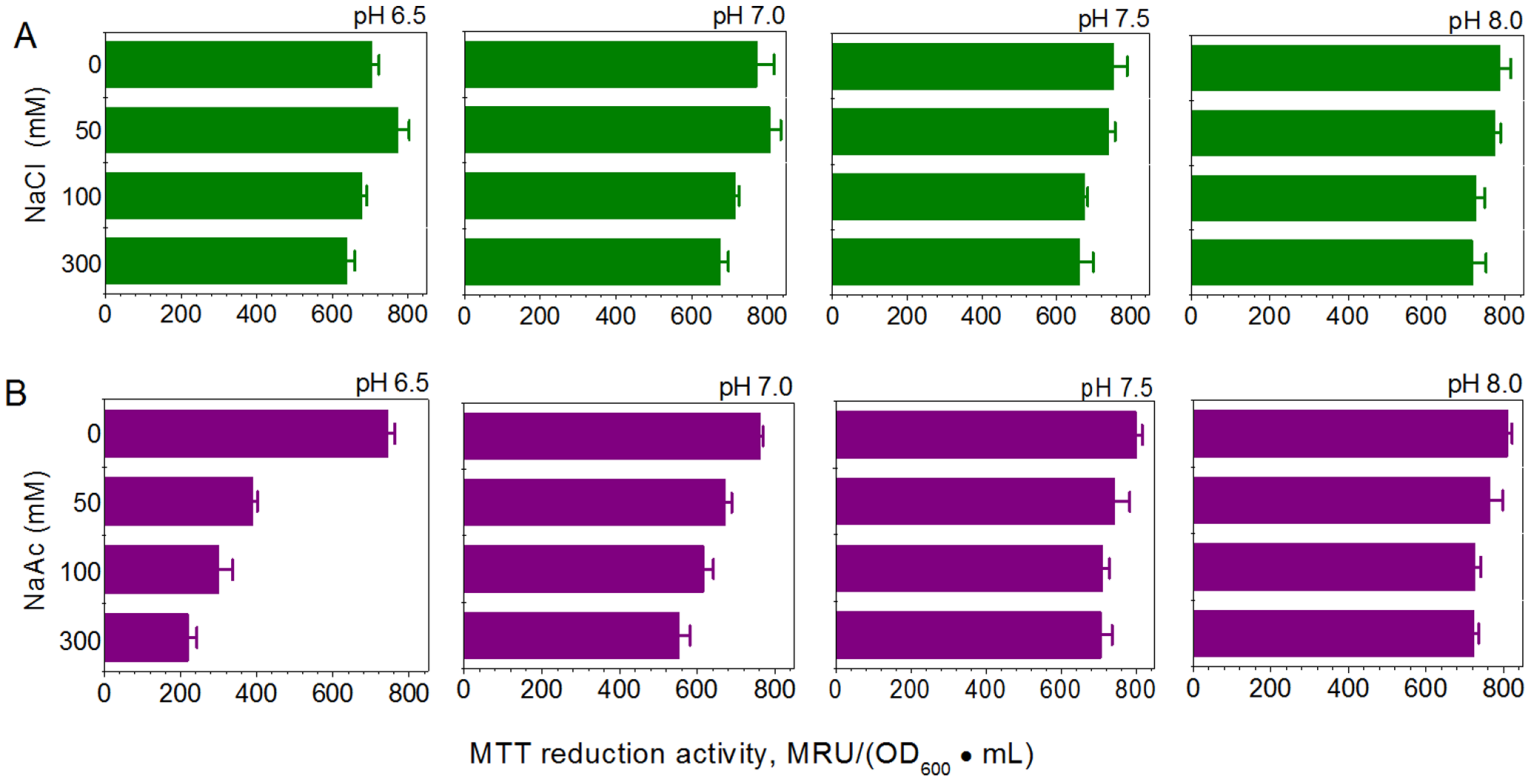
Effects of pH on the MTT-reducing activity of the *E. coli* cell membrane under acetate stress. *E. coli* BL21 (DE3) cells were investigated on 0, 50, 100 and 300 mM NaCl (A) or NaAc (B) in BYT-glycerol medium at pH 6.5, 7.0, 7.5 and 8.0.

For BYT-glycerol medium containing no additional NaCl or NaAc, electron production by the *E. coli* cell membrane remained at a level of 760±35 MRU/(OD_600_ • mL) when the medium pH decreased from 7.5 to 6.5 (Figure 8A and 8B). For BYT-glycerol medium containing 50–300 mM NaCl, when the pH shifted from 7.5 to 6.5, the electron production decreased slightly, by a drop of less than 10% (Figure 8A). For example, at 300 mM NaCl, the activities were estimated to be 665±36 at pH 7.5 and 638±21 MRU/(OD_600_ • mL) at pH 6.5. In contrast, for the medium contained 50–300 mM NaAc, the shift from pH 7.5 to 6.5 caused a sharp drop in electron reduction, by approximately 55–75%, depending on acetate concentrations (Figure 8B). In the presence of 300 mM NaAc, the activities were estimated to be 706±30 MRU/(OD_600_ • mL) at pH 7.5 and 220±23 MRU/(OD_600_ • mL) at pH 6.5, a drop of 69%. Our results show that in the presence of acetate, a one-unit drop in pH from 7.5 to 6.5 causes a drop in bacterial MTT activity, although pH 7.5 and 6.5 are both suitable for cell growth in the BYT-glycerol medium without any acetate (Figure 2).

## Discussion

Accumulation of acetate has long been an issue for recombinant protein production by *E. coli* cells. Initial efforts focused mainly on reducing acetate excretion, generally by deleting genes in the pathways of glucose uptake and acetate formation, or controlling the glucose feed rate [5], [6]. Recently, improvements in acetate tolerance by genetic strategies and medium supplementation with certain amino acids and pyrimidines have shown much promise. The overall objective of our study was to use the simple approach of an alkaline pH shift to increase the acetate tolerance of *E. coli* BL21 (DE3) cells, a popular strain currently used in recombinant protein expression.

### Proposal: a shift to alkaline pH

**Figure 9:**
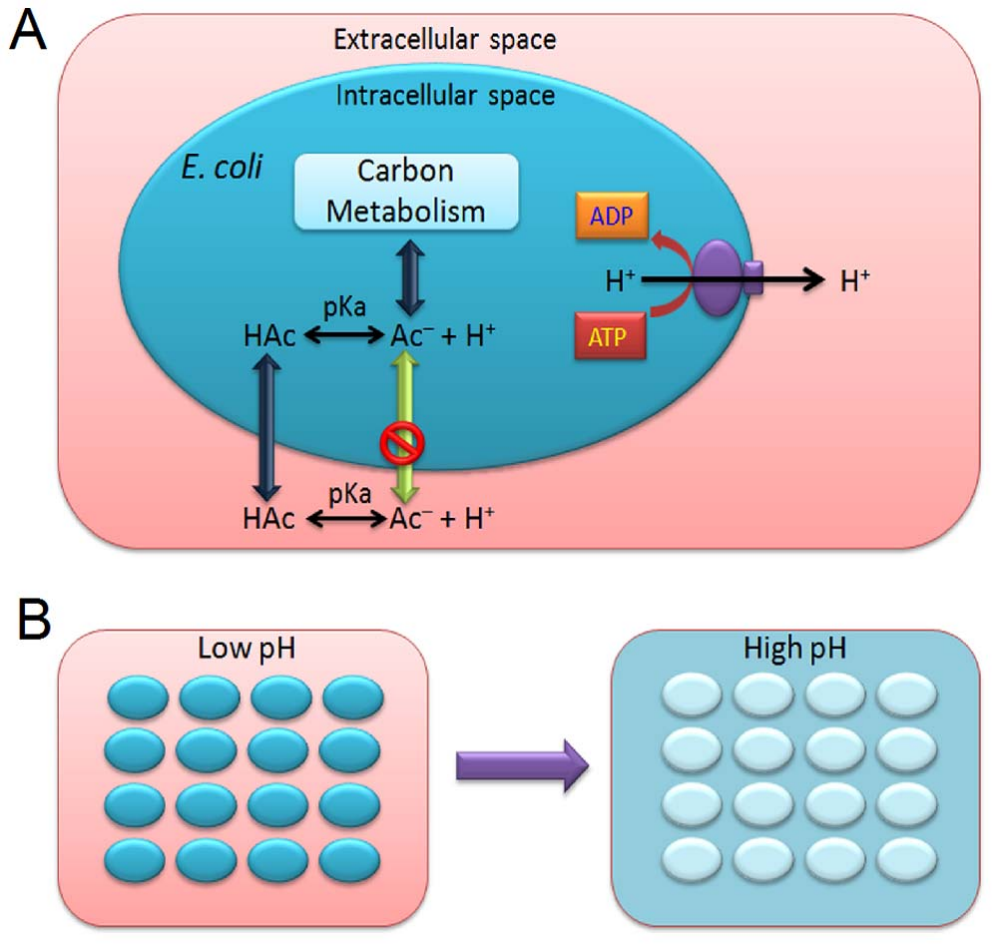
A schematic representation of the decrease in concentration of extracellular harmful acetic acid molecules (HAc) and intracellular acetate ions (Ac^–^) produced by an alkaline pH shift. A) Diagram to show HAc formation from Ac^–^ and diffusion across the cell membrane as an uncoupler. Ac^–^ cannot permeate the *E. coli* cell membrane. B) Release of intracellular acetate by an alkaline extracellular pH shift from 6.5 to a higher pH. Dark blue indicates higher intracellular acetate concentrations. pKa, the dissociation constant of acetic acid.

The anion Ac^–^ always accompanies the molecule HAc and their relative proportions may change with adjustment of the medium pH. HAc can diffuse freely across cell membranes, resulting in the collapse of the transmembrane pH gradient, and dissociates intracellularly into Ac^–^, causing an Ac^–^ accumulation if the intracellular pH is higher than the medium pH (Figure 9A) [3], [14], [32]. Our proposal in this study is that, in the case of *E. coli* BL21 (DE3) cells, an alkaline medium pH (7.5 to 8.5) reduces the concentration of toxic extracellular HAc molecules and thus reduces the intracellular Ac^–^ accumulation, which may help to alleviate growth inhibition, increase cell viability and preserve the expression of recombinant proteins (Figure 9B).

### Under acetate stress: a one-unit decrease in pH from 7.5 to 6.5 caused a drop in *E. coli* BL21 (DE3) cell growth and protein expression

Although the adverse effects of acetate have been widely reported, the results of available studies on acetate tolerance in *E. coli* in terms of cell growth and protein expression have been disparate and sometimes seemed inconsistent [30], [33], [34]. Jensen and Carlsen used *E. coli* MC1061, a derivative of *E. coli* K12, to produce a human growth hormone precursor (MAE-hGH) and found that, in HKSII medium (pH 7.2) containing 5 g/L glucose, acetate addition resulted in inhibition of cell growth at concentrations from 6 g/L (100 mM), but a significant decrease in the specific production rate of MAE-hGH at concentrations from 2.4 g/L (40 mM) [30]. Ko and coworkers used *E. coli* F-122 to express HIV582-β-galactosidase fusion protein in a modified M9 minimum medium (pH 7.0) supplemented with L-tryptophan, L-leucine, L-proline and vitamin B1 [33]. They found that the presence of acetate up to 3 g/L (50 mM) produced little inhibition of β-galactosidase production and suggested that acetate may not act as a specific inhibitor of protein production, at least for the concentrations tested [33].

The most commonly used pH for *E. coli* cell cultivation and recombinant protein expression is around 7.0, ranging from 6.5 to 7.5. Although pH 6.5 and pH 7.5 are both suitable for cell growth and protein expression in a medium without acetate, there may be a great difference in the presence of acetate. Our results showed that pH 7.5 significantly reduced the intracellular acetate concentration and improved the specific growth rate (Figure 2 and 3). In the presence of 300 mM NaAc, the expression levels of GST, CYP and GFP decreased at pH 6.5, though by different levels, but the expression levels were preserved at pH 7.5 (Figure 6). Further experiments showed that at alkaline pH (7.5 to 8.5) cell growth was well supported and there was little inhibition of the expression of GST, CYP and GFP (Figure 4 and 5). In addition, at 50–300 mM NaAc, bacterial MTT-reducing activity decreased at pH 6.5 by 50–75% but recovered at pH 7.5∼8.0 (Figure 8).

### NaAc at 300 mM: effect of uncoupling HAc and intracellular acetate anion accumulation

It is a long-held view that the undissociated form of the weak acid HAc is an uncoupler and is able to cause a collapse of the transmembrane ΔpH (the proton-motive force across the cytoplasmic membrane) [3]. However, the presence of uncoupler HAc does not negate the idea that Ac^–^ anions will accumulate intracellularly in response to the ΔpH, especially at low medium pH. Russell argued that the uncoupler theory did not explain well why some bacteria tolerate volatile fatty acids even when the extracellular pH is low [32]. He grew *Streptococcus bovis* JB1, an acid-tolerant ruminal bacterium, at low pH values (6.7 to 4.5) and found that 100 mM acetate had little effect on the growth rate or proton motive force across the cell membrane [32]. Further results showed that the strain allowed its intracellular pH to decrease and maintained a relatively constant pH gradient across the cell membrane, thus preventing the intracellular accumulation of large amounts of Ac^–^ [35], [36].

To date, the side effects of acetate on cell growth and recombinant protein expression have been widely studied, but at concentrations far lower than 300 mM. In our experiments, up to 300 mM acetate in the form of NaAc was used and 300 mM NaCl was chosen as the control to mimic the salt stress from NaAc. The results showed that the salt stress of 300 mM NaCl at pH 6.5 and 7.5 had little effect on *E. coli* cell growth, recombinant protein expression or cellular MTT-reducing activity (Figure 2, 5 and 8). In the case of 300 mM NaAc, according to the Henderson-Hasselbalch equation, the concentration of the toxic molecule HAc was estimated to be 0.53 mM at pH 7.5 and 5.24 mM at pH 6.5 in aqueous solution. In addition, an extracellular alkaline pH of more than 7.5 also helps in the excretion of intracellular acetate, due to the permeability of the molecule HAc and impermeability of Ac^–^ across the *E. coli* cell membrane (Figure 9A). Suppose that the *E. coli* cells were able to maintain a constant cytosolic pH of around 7.5 and the cell membrane was a chemically pure membrane that only allowed free diffusion of the uncoupler HAc: at equilibrium, the concentrations of HAc inside and outside the cell would be the same and the intracellular acetate concentrations would be approximately 3000 and 300 mM in media at pH 6.5 and pH 7.5, respectively. However, the above theory sounds reasonable chemically, but not physiologically. Our results for pH 6.5 are not consistent in quantity with the results from the above hypothesis. In the 40-min incubation on 300 mM NaAc, the intracellular acetate was estimated to be 576 mM at pH 6.5, approximately two-fold that of extracellular acetate. This is due at least in part to an intracellular pH decrease resulting from the dissociation of intracellular HAc suggested by Russell [35], [36], Slonczewski et al. [37] and Roe et al. [14]. In any case, a shift to alkaline pH is able to decrease both the concentration of HAc and the intracellular acetate accumulation and, thus, reduce their toxicity in *E. coli* cells.

*E. coli* is currently one of the suitable microbial hosts for biofuels and biochemicals production from cellulosic and hemicellulosic sugars [38]–[41]. Acetate may be produced by *E. coli* cells through fermentation on such sugars and chemical degradation of lignocellulosic biomass [1], [2], [42]. HAc, as a volatile molecule, is able to diffuse freely through *E. coli* cell membranes and form more Ac^–^ anion on the side with higher pH values [3], [35], [36]. Our tests on *E. coli* BL21 (DE3) shows that an appropriate increase in extracellular pH drove the intracellular acetate out of cells, changed the intracellular toxicity of acetate into the extracellular osmotic pressure of Ac^–^, and thus, improved the resistance of cells to acetic acid (Figure 1 and Figure 9). Alleviation of the acetate-caused cell growth inhibition by alkaline pH shift from pH 6.5 to pH 8.0 was also observed in the case of *E. coli* Origami (DE3) and DH5α (Figure 7B and 7C). The above approach of HAc export may be helpful in improving acetate resistance of other *E. coli* strains in the production of biofuels and biochemicals from sugars.

In conclusion, in this study we addressed the effects of the crucial factor of medium pH on the performance of *E. coli* cells grown on acetate at high concentrations. Under the stress of 300 mM acetate, the intracellular acetate accumulation was greatly reduced at pH 7.5 compared with pH 6.5; moreover, at pH 7.5 the cellular growth improved and the expression of recombinant proteins GST, CYP and GFP was restored. Further experiments demonstrated that alkaline pH up to 8.5 had little effect on the expression of GST, CYP and GFP. Thus, we suggest an approach of cultivating *E. coli* BL21 (DE3) at pH not less than 7.5 to combat acetate stress. However, it also should be noted that sterilization of the complex medium at alkaline pH tends to cause cell growth inhibition (data not shown), and thus it is necessary to adjust the medium pH using NaOH solution on inoculation. In brief, the proposed strategy of an alkaline pH shift introduces an efficient paradigm for cultivating *E. coli* cells, which was shown to improve the expression of recombinant proteins and may help in the production of biofuels and biochemicals from lignocellulosic hydrolysates containing high concentrations of acetate.
